# Light and Enzymatic Cooperative Response in Supramolecular Fibers: A Synergistic Strategy for Potential Drug Delivery Applications

**DOI:** 10.1021/acs.biomac.6c00354

**Published:** 2026-04-17

**Authors:** Mónica Martínez-Orts, Edgar Fuentes, Yeray Gabaldón, José Augusto Berrocal, Lorenzo Albertazzi, Silvia Pujals

**Affiliations:** † Department of Biological Chemistry, Institute for Advanced Chemistry of Catalonia (IQAC–CSIC), Barcelona 08034, Spain; ‡ Institute of Chemical Research of Catalonia (ICIQ), Avda. Països Catalans 16, Tarragona 43007, Spain; § Catalan Institution of Research and Advanced Studies (ICREA), Pg. Lluís Companys 23, Barcelona 08010, Spain; ∥ Institute of Complex Molecular Systems, Eindhoven University of Technology, MB Eindhoven 5600, The Netherlands

## Abstract

Supramolecular Polymers (SPs) can undergo reversible self-assembly in response to internal or external stimuli. Design strategies based on the benzene-1,3,5-tricarboxamide (BTA) core are gaining increasing attention. In this study, we describe a BTA amphiphile that self-assembles into supramolecular fibers in water and is able to respond to both light and enzymatic activity. An azobenzene moiety (AZB) and Gly-Phe-Leu-Gly amino acid sequence (GFLG) were incorporated into the BTA monomer skeleton to build three identical wedges that respond to light and Cathepsin B activity. The synergistic application of two orthogonal stimuli enabled modulation of the assembly of BTA-AZB-GFLG fibers through the cooperative response to stimuli. These findings provide new insights into the use of SPs for future drug delivery applications.

## Graphical abstract

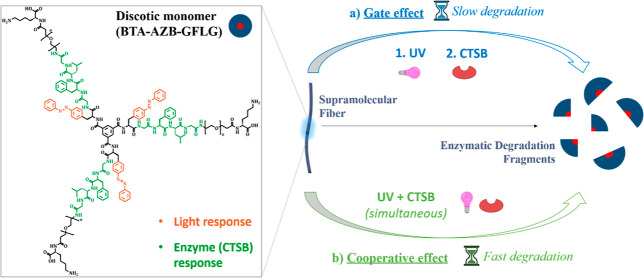

## Introduction

Supramolecular polymers (SPs) have gained growing interest over the last years,^1–6^ evolving from early applications, like lithographic printing, to current uses in fields ranging from electronics to medicine. Inspired by nature,^7,8^ SPs rely on noncovalent interactions, including hydrogen bonding and π–π stacking, which stabilize one-dimensional polymeric assemblies.^9^ Due to the noncovalent nature of SPs, the disassembly can be triggered in response to external stimuli or environmental changes.^10^ Light, ultrasound, or electric fields are among the most studied stimuli that can be applied from external sources. In contrast, physiological changes in the temperature, pH, redox species, or enzymatic activity are caused by cancer and other pathologies. These altered factors can be exploited as internal stimuli for responsive systems. Especially in cancer, enzymes are investigated as potential triggers for controlled drug activation or release, since their expression and activity are significantly altered in the presence of tumors.^11–16^ On the other hand, light is a minimally invasive stimulus that can be applied to achieve high spatial resolution and temporal control.^17,18^

In this work, we design an SP able to respond to one internal (enzyme) and one external (light) stimulus and study the assembly state controlled by these synergistic cues.

The dynamic and tunable features of SPs, along with their ability to respond to diverse stimuli,^19,20^ offer valuable insights into their tailored design for biomedical applications.^21,22^ In recent decades, considerable efforts^23,24^ have focused on optimizing the design of such dynamic supramolecular systems with the aim of combining structural robustness with biological responsiveness, two key aspects in biomedicine. Nevertheless, achieving the appropriate release profile for stimuli-responsive SPs in drug delivery has remained poorly explored. We recently reported, for the first time, the controlled release of a hydrophobic ligand (iperoxo-axo, IoA) encapsulated into the inner pocket of a responsive SP based on the benzene-1,3,5-tricarboxamide (BTA) core.^25^ BTAs have been widely exploited in supramolecular chemistry^26–29^ because of their ability to self-assemble into supramolecular fibers (in water). This core enables the construction of *C*_3_-symmetric arrays that drive self-association into columnar π-stacked aggregates, stabilized by 3-fold hydrogen bonding,^30^ as extensively demonstrated by the Meijer group.^31–38^ Their pioneer studies revealed that BTA derivatives can also give place to functional supramolecular assemblies in aqueous environments by decorating the aromatic core with designed chains that promote water solubility.^39^

On account of its synthetic modularity, the BTA core allows us to build smart systems with three identical wedges designed to imprint stimuli-responsiveness. In this field, the development of multiresponsive SPs offers a promising strategy to fine-tune the transport and release of drugs.^40–44^ Previously, we developed a novel design for water-soluble supramolecular fibers that could independently respond to four different stimuli.^45^ The BTA-based discotic amphiphile contained specific responsive blocks: a non-natural azobenzene amino acid (light response), an octa­(ethylene glycol) amino acid (PEG_8_, thermal responsivity), and a C-terminal lysine (dual pH and ionic strength response). The different modules were strategically placed to impart hydrophobicity of the innermost block, which is shielded by a hydrophilic layer that also grants flexibility and water solubility. Building on this design, here, we additionally incorporate an enzyme-responsive module (Gly-Phe-Leu-Gly, GFLG) to achieve selectivity for cancer cells. GFLG, evaluated as a cleavable spacer in clinical trials for the delivery of drugs,^46–48^ is a well-established substrate for Cathepsin B (CTSB). The expression of this cysteine protease is often altered in various cancer cells^49,50^ (brain, lung, prostate···), and it also regulates key pathological processes of related brain disorders,^51–53^ like neurodegeneration of Alzheimer’s Disease. However, CTSB is largely found in human tissues and cells, where it is involved in several intracellular functions, or as a key component in the extracellular space.^54–56^ Thus, this ubiquitous localization also hinders the selective release of drugs targeted by the CTSB-based DDSs.

To overcome this issue, here, we establish the synergistic combination of two different stimuli to modulate the response of the system to the enzyme. On one hand, the GFLG sequence secures the sensitivity to CTSB, while the azobenzene moiety (AZB) imparts light-responsiveness. The structure of the BTA-AZB-GFLG monomer is shown in Figure 1. The discotic amphiphile was designed to self-assemble as a CTSB-sensitive system that triggers the enzyme response only after the application of two consecutive stimuli. To achieve that, the GFLG sequence was placed close to the inner core to avoid easy access to CTSB. Introducing the GFLG sequence in this position should also protect the hydrophobic core and further promote self-assembly by additional H-bonding.

**Figure 1:**
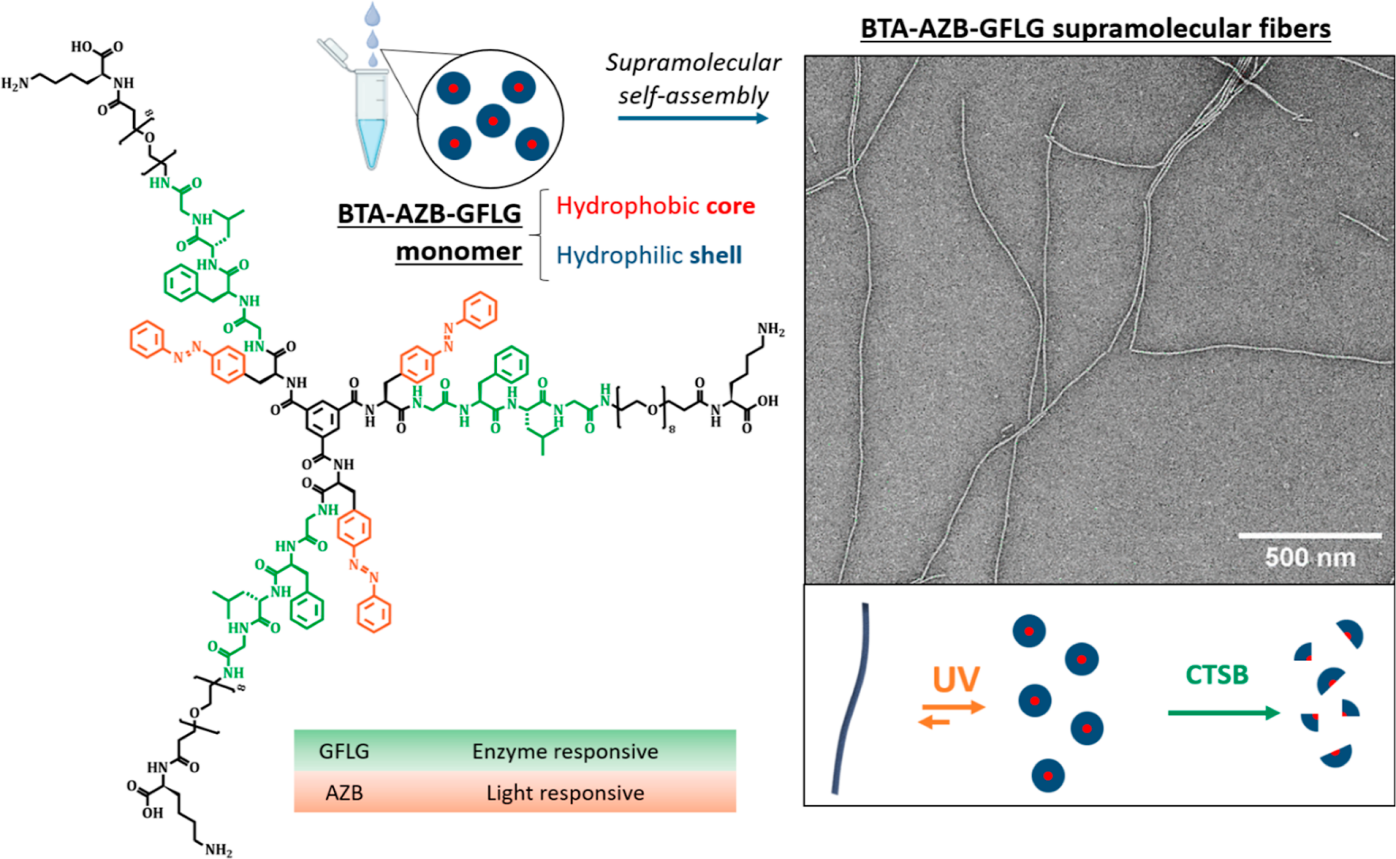
(Left) BTA-AZB-GFLG monomer. (Right) TEM micrograph and mechanistic proposal of cooperative stimuli response.

## Experimental Section

### Materials

All solvents (acetonitrile—ACN, chloroform—CHCl_3_, dichloromethane—DCM, *N*,*N*-dimethylformamide—DMF, and dimethyl sulfoxide—DMSO) were obtained from commercial sources (Carlo Erba, Merck, and PanReac). Ultrapure water was obtained from a Milli-Pear system from Merck.

Fmoc-Lys­(Boc)-OH and 2-chlorotrityl chloride resin were purchased from Iris Biotech. Fmoc-octa­(ethylene glycol) amino acid and HBTU were purchased from ChemPep. DIEA, piperidine, formic acid, and benzene tricarbonyl chloride were obtained from Sigma-Aldrich. Fmoc-Gly-OH was purchased from Peptide Institute Inc., Fmoc-l-Phe-OH was purchased from ChemPep, and Fmoc-l-Leu-OH was purchased from Iris Biotech.

l-Phenylalanine-4′-azobenzene was synthesized by Dr. José Augusto Berrocal, following the procedure reported before.^57,58^

Cathepsin-B from the bovine spleen (≥10 U/mg protein) was obtained from Merck.

### Chemical Synthesis

The strategy employed to synthesize the BTA-AZB-GFLG monomer, Scheme S1, consisted of the use of solid-phase peptide synthesis (SPPS) to grow the responsive wedge, followed by solution-phase coupling to the BTA core.

(i) Solid-Phase Peptide Synthesis
Resin swelling. Trityl chloride resins are among the most widely used solid supports for the SPPS procedure.^59^ Here, we employed 2-chloro-trytil chloride resin (1.6 mmol/g) that was transferred to a reaction syringe and swelled with the appropriate solvents: DCM (15 mL ×5 + 15 mL for 1 h), DMF (15 mL ×5 + 15 mL for 30 min), DCM (15 mL ×5 + 15 mL for 1 h).
Loading (i). The attachment of the first residue to the resin support was performed. This is one of the most critical steps in SPPS. A 5 mL solution of DCM containing 1 equiv of Fmoc-Lys­(Boc)-OH and 3 equiv of DIEA (*N*,*N*-diisopropylethylamine) was coupled to the resin for 1 h, with manual agitation for the first 5 min. Subsequently, to cover the unreacted sites, 1 mL of MeOH was allowed to react for 10 min. Finally, the mixture was washed with DCM (15 mL ×3), DMF (15 mL ×3), and DCM (15 mL ×3).
Fmoc deprotection (ii, iv, vi, viii). After each coupling, the Fmoc group from the *N*-terminus of the resin-coupled peptide is removed under mild conditions. The resin was first treated with piperidine in DMF at 20% v/v for 10 min (×2). Later on, it was washed with DMF (15 mL ×3) and DCM (15 mL ×5).
Octa­(ethylene glycol) coupling (iii). 1.5 equiv of Fmoc-octa­(ethylene glycol)-OH was preactivated with 1.5 equiv of HBTU (hexafluorophosphate benzotriazole tetramethyl uronium) and 4.5 equiv of DIEA, in DCM:DMF (9:1). The mixture was placed into the syringe, shaken for 15 h, and stirred for 5 min every hour during the initial 3 h. Next, the syringe contents were filtered and washed with DCM (15 mL ×3), DMF (15 mL ×3), and DCM (15 mL ×3).
Successive coupling of Nα-protected building blocks (iii–viii). 1.5 equiv of the corresponding amino acid residue (Fmoc-octa­(ethylene glycol)-OH, Fmoc-Gly-OH, Fmoc-Phe-OH, Fmoc-Leu-OH, Fmoc-Gly-OH, and Fmoc-Azo-OH) was first preactivated with 1.5 equiv of HBTU and 4.5 equiv of DIEA, in DCM:DMF (1:1). The mixture was placed into the syringe, shaken for 15 h, and stirred for 5 min every hour during the initial 3 h. Next, the syringe contents were filtered and washed with DCM (15 mL ×3), DMF (15 mL ×3), and DCM (15 mL ×3).
Cleavage from resin (ix). The resin was treated with 1% TFA v/v in H_2_O for 2 min, filtered, and dried by N_2_ steam. This procedure was performed twice. The final peptide chain was purified by DCM:H_2_O phase extraction and vacuum-dried. The corresponding GFLG wedge was isolated.
(ii) Solution Synthesis
Core coupling (x). 1 equiv of GFLG wedge (22.4 mg, 17.3 μmol) and 3 equiv of Et3N (7.23 μL, 51.9 μmol) were dissolved in CHCl_3_ and cooled to 0 °C under stirring. Meanwhile, 0.25 equiv of 1,3,5-benzenetricarbonyl trichloride (1.15 mg, 4.3 μmol) was dissolved in CHCl_3_ and was added dropwise to the mixture of GFLG wedge and Et3N under continuous stirring at 0 °C.

After 15 min, the reaction was removed from the ice bath, and it was stirred another 12 h at room temperature. The reaction mixture was concentrated in vacuo (to remove CHCl_3_) and further dissolved in H_2_O:ACN (1:1) and vacuum-dried.

Boc removal (xi). BTA-AZB-GFLG was treated with TFA:H_2_O:TIPS (triisopropyl silane) 95:2.5:2.5 for 1 h at room temperature. The product was dried by N_2_ steam and dissolved in H_2_O:ACN (1:1) for further purification by reversed-phase HPLC and vacuum-dried yielding BTA-AZB-GFLG (30.1%).

### Instrumentation

(i) Reversed-phase HPLC semipreparative chromatography

The purification of BTA-AZB-GFLG was performed using a Waters Alliance 2695 separation module, equipped with a Waters 2489 UV–vis detector that allowed us to follow the separation at 280 and 330 nm. Milli-Q water 0.1% HCOOH and ACN 0.1% HCOOH were used as eluents with a gradient of 30–70% (ACN-Milli-Q) applied for 30 min. The column used was LC Column Peptide XB-C18 (5 μm, 150 × 21.2 mm). The mobile phase consisted of acetonitrile 0.1% HCOOH (A) and Milli-Q water 0.1% HCOOH (B). The elution method was carried out at a runtime of 25 min and flow rate of 10 mL/min.

(ii) Ultrahigh Performance Liquid Chromatography and detection by means of Time of Flight Mass Spectrometry (UPLC-TOF MS)

BTA-AZB-GFLG was identified by its retention time and exact mass by UPLC-TOF MS analysis. The UPLC system (Select Series Cyclic IMS, Waters) was equipped with a PDA detector (Acquity Premier PDA). Column used: Acquity Premier BEH C18 (1.7 μm, 2.1 × 100 mm). Mobile phase: Milli-Q water 0.1% HCOOH (A) and acetonitrile 0.1% HCOOH (B). The elution method was performed at a runtime of 12 min and a flow rate of 0.4 mL/min.

(iii) High-resolution reversed-Phase Liquid Chromatography (HPLC) coupled to Mass Spectrometry (MS)

To monitor BTA-AZB-GFLG purification and enzymatic degradation, HPLC-MS chromatograms were recorded on an Acquity Arc multidimensional liquid chromatography (MDLD) system from Waters. This system was equipped with a PDA (PhotoDiode Array) detector (Waters 2998), an Evaporative Light-Scattering Detector (ELSD, Waters 2424), and a Mass Spectrometer SQ Detector (Single Quadrupol). Column used: Zorbax RR Exent C18 (3.5 μm particle size, 2.1 × 50 mm). Mobile phase: Milli-Q water 0.05% HCOOH (C) and acetonitrile 0.05% HCOOH (D). The elution method was carried out at a runtime of 7 min and a flow rate of 0.7 mL/min. MassLynx version 4.2 (Waters, Milford, MA) supported the acquisition and analysis of HPLC-MS data.

(iv) UV–vis Spectroscopy

The absorbance spectra were acquired with a UV–vis Agilent Cary 60 instrument using a baseline correction method. High precision ultramicro quartz cuvettes (105-200-85-40, Hellma) were purchased by LineaLab. The spectra were performed at room temperature at a scan rate of 300,000 nm/min.

(v) Circular Dichroism (CD)

The experiments were acquired on a Jasco J-1500 spectrometer, and quartz cells with a path length of 10 mm were used. By using baseline correction, the spectra were recorded continuously between 500 and 200 nm at every 0.1 nm, with a scanning speed of 100 nm/min. The temperature was kept at 20 °C and 3 accumulations were measured.

(vi) TEM

A JEOL JEM 1010 microscope (operating at 80 kV) was used to acquire TEM images. Samples were deposited on C-only grids (200 mesh) that were preactivated with air glow discharge for 30 s in a vacuum chamber (0.1 mbar). The protocol for sample deposition consisted of

1. Deposition of 1 drop of sample for 1 min.
2. Washing with Milli-Q water (1 drop for 10 s, ×3).
3. Negative staining with 1 drop of uranyl acetate 2% for 1 min.
4. The excess solution was blotted using filter paper, and the grids were dried in the desiccator.

ImageJ software (National Institutes of Health, Bethesda, Maryland, USA)^60^ was used to process microscopy images.

### Supramolecular Assembly

To trigger the self-assembly, an aliquot of the concentrated stock solution was dispersed into Milli-Q water to a final concentration of 25 μM. The mixture was annealed at 70 °C for 1 h and then equilibrated at room temperature for 24 h.

### Enzyme and Light Response

(i) Irradiation setup

Thorlabs M365LP1-C5 was used as the UV source at 365 nm. Irradiations were performed at 100% of the LED intensity (1000 mA). To work in dark conditions, a custom black box was handmade, maintaining a distance of 15 cm between the sample and the light source in all cases. The sample was placed in a heating thermoshaker instrument to maintain the same conditions also in the continuous irradiation experiment.

(ii) Enzymatic Response

The solvent used for the dilution of the enzyme and the preparation of supramolecular fibers was an aqueous buffer for achieving the optimal activity. The required buffer conditions must reproduce those found in the lysosomes, where CTSB is located,^61^ i.e., reducing environment (provided by DTT reagent) and weak acidity (HCl 2 M was used to adjust the pH to 5.0 ± 0.1). Thus, the buffer, prepared in Milli-Q water, contained sodium acetate (50 mM), dithiothreitol (DTT, 30 mM), and ethylenediaminetetraacetic acid (EDTA, 1 mM). The first step consists of the activation of Cathepsin B (CTSB, 43.7 u/mL) by previous incubation at 37 °C for 20 min.^62^ BTA-AZB-GFLG supramolecular fibers (25 μM, in the same buffer conditions) were also preheated at 37 °C. Then, 20 μL of CTSB was added to BTA-AZB-GFLG supramolecular fibers (the final concentration of CTSB was 3.8 u/mL). The enzymatic cleavage of the GFLG sequence by the action of CTSB was monitored with HPLC-MS and TEM microscopy.

## Results and Discussion

### BTA-AZB-GFLG Fiber Characterization

The responsive wedge incorporating AZB, GFLG, PEG_8_, and C-terminal Lys was first synthesized by solid phase peptide synthesis and then coupled to the BTA core in solution (Figure S1 and Scheme S1) yielding the final trisubstituted molecule BTA-AZB-GFLG. The monomer was molecularly dissolved in DMSO and then injected into an aqueous buffer solvent (optimal conditions for CTSB activity) to trigger the self-assembly. Transmission Electron Microscopy (TEM) images revealed the formation of 1D fibers with a uniform width of 9.5 ± 0.4 nm and varying lengths (30% of the fibers below 500 nm, and almost 70% between 500 nm and a few micrometers) (Figures 1 and S2). The helical chirality of the supramolecular self-assemblies was studied by Circular Dichroism (CD), which revealed a series of strong Cotton effects centered at 325 nm (corresponding to AZB): a negative peak at 345 nm, and a positive peak at 290 nm (Figure S3).^63–65^ A series of less intense Cotton effects related to the BTA core were also detected at 232 nm, with a weak positive peak at 227 nm and a negative peak at 255 nm. The aggregation of BTA-AZB-GFLG fibers was also evaluated by UV–vis spectroscopy (Figure S4). The absorption peak associated with the *E*-AZB exhibited a blue shift of 13 nm upon going from the nonassembled state (λ = 328 nm) to the assembled state (λ = 315 nm).^66^

### Cathepsin B Response of BTA-AZB-GFLG Fibers

Then, we evaluated the enzyme response of BTA-AZB-GFLG fibers in the presence of CTSB at different incubation times. As hypothesized, even after 96 h of incubation with CTSB, 89.1% of the monomers remained intact, as quantified by HPLC-MS (Figure S5). These results were supported by TEM images, which revealed a minimal enzymatic response (Figure 2). Minor changes were observed by TEM as the analysis of the images revealed the appearance of new small spherical structures (with a diameter of less than 10 nm) after the enzymatic incubation. In addition, the population of shorter fibers (<200 nm) diminished after 96 h of enzyme incubation (Table S1). To rule out the possibility that these spheres had originated from CTSB, control TEM images of the enzyme alone were also acquired (Figure S6). Congruously, no spherical structures were detected. This assay led us to conclude that a majority of the fibers did not respond to CTSB after a single stimulus (i.e., exposure to the enzyme). We attribute this behavior to the incorporation of the three additional aromatic rings derived from phenylalanine (Phe), which enhances hydrophobicity and columnar stacking of the fibers via π–π interactions, consequently hindering enzymatic cleavage of the GFLG sequence (Figure S7). We thus hypothesized that the tailored application of light on the AZB moiety would disrupt the strong assembly of the fibers,^45^ liberating free monomers that would then undergo enzymatic cleavage by CTSB. The use of photoswitchable motifs in the molecular design of smart materials attracts special attention,^67–75^ particularly in the field of drug delivery, where the development of light-responsive nanocarriers has been widely explored.^45,76–78^

**Figure 2:**
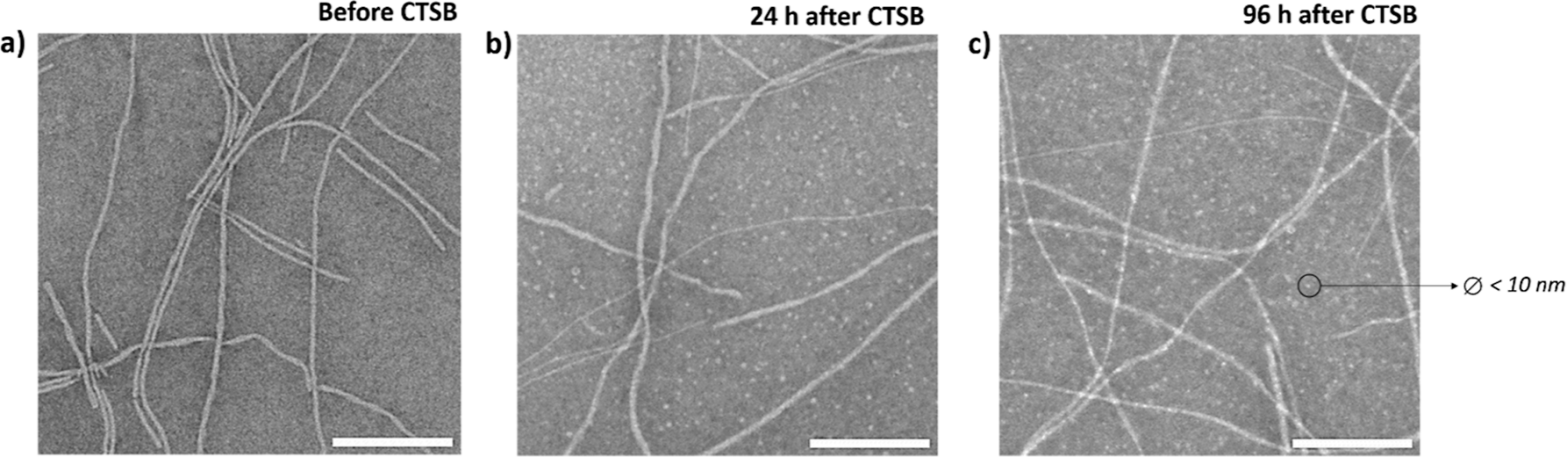
TEM images of BTA-AZB-GFLG fibers (25 μM) before CTSB (3.8 U/mL) (a) and after 24 h (b) and after 96 h (c) of incubation. Scale bar: 200 nm.

### Light Response of BTA-AZB-GFLG Fibers

The light response of BTA-AZB-GFLG (Figure S8) was tested by irradiation with 365 nm light (UV light). UV–vis absorbance spectra of BTA-AZB-GLFG fibers (Figure S9) showed a mild *E*–*Z* photoisomerization (44.7%) compared to the nonassembled monomer (with a 99.9% composition of *E*-configured AZB). The strong fibrillary packing of BTA-AZB-GFLG assemblies could explain the mild response to UV light. The photoresponse of supramolecular fibers was also confirmed by CD and TEM microscopy (Figure S10). The mild response to light stimulus, as noted above, was also confirmed by a very slight decrease in the CD signal. TEM images further revealed that self-assembly persisted after UV irradiation. However, we observed the disappearance of fibrillar aggregates with a length in the range of 401–2200 nm, producing an increase in the population of fibers with a length below 400 nm.

### Dual Stimuli Response: Gate Effect

Next, we sought to combine both light irradiation and CTSB incubation to obtain a cooperative effect over fibrillary degradation. To demonstrate that the enzyme response of BTA-AZB-GFLG fibers is triggered by dual-stimuli-responsiveness, we first irradiated BTA-AZB-GFLG fibers with ultraviolet light for 10 min. Immediately after, the irradiated fibers were incubated with CTSB. HPLC-MS experiments were acquired at different times (Figure 3a,b). The results showed that UV-irradiated monomer achieved almost complete enzyme response (0.1% of remaining fibers) after 96 h of incubation with CTSB (Figure S11). Fibrillar degradation significantly increased compared to the control experiment (95% of untouched monomer after 96 h of incubation with CTSB) in which the system was not irradiated with UV light prior to enzyme treatment (referred to Figure 2). These results were corroborated by TEM microscopy (Figure 3c). Furthermore, we tested the thermal stability of the Z-AZB in BTA fibers during 96 h at 37 °C by HPLC-MS (Figure S12). The results showed an absence of monomer fragmentation, confirming that the degradation observed in the presence of CTSB was primarily enzymatic.

**Figure 3:**
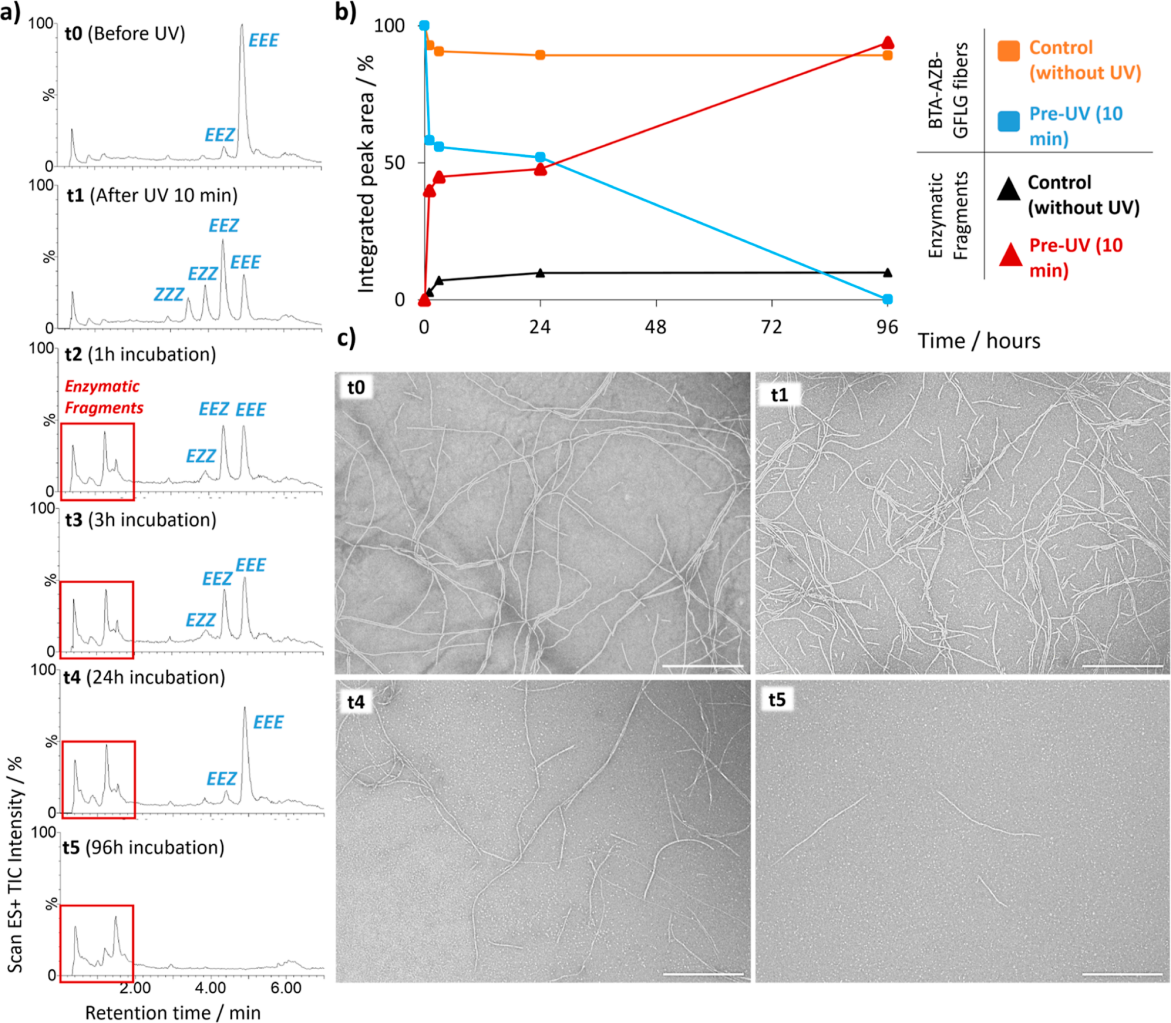
Enzyme response of BTA-AZB-GFLG (25 μM) to CTSB (3.8 U/mL) with pre-UV irradiation (gate effect). HPLC-MS chromatograms (a) and integrated peak areas (b) following the enzymatic degradation. (c) Representative TEM micrographs (scale bar: 500 nm).

Taken together, these observations support the hypothesis that irradiation with UV light prior to CTSB addition (gate stimuli response) favors the enzymatic degradation of the BTA-AZB-GFLG monomers. This conclusion is supported by additional experiments in which the dual responsive (i.e., light + enzyme) behavior was reproduced in the initial assembly state. These experiments were performed via rapid formulation of the fibers without carrying out any thermal annealing to stabilize the longer aggregates. The BTA-AZB-GFLG monomer was first irradiated with UV light for 10 min in DMSO and then immediately injected into the buffer solution that already contained the CTSB enzyme. HPLC-MS results revealed a complete enzyme response in the initial assembly state after 1 h of incubation, in stark difference to the control experiment, with no pre-UV irradiation (Figure S13). These results suggest that steric effects and interactions within the assembly may hinder photoisomerization of the AZB motif in BTA-AZB-GFLG fibers. Indeed, we did not allow fibers to stabilize and to form large and tightly packed assemblies in the case of the initial assembly state, an idea that has been previously reported in the context of other supramolecular assemblies.^77,79–81^ Building on our experimental results, we propose that UV irradiation shifts the self-assembly equilibrium toward a larger molar fraction of nonassembled monomers, which are then rapidly hydrolyzed by CTSB. Hence, the hydrolyzed fragments likely do not participate in the equilibrium of the supramolecular fibers. These hydrolyzed fragments or a high amount of monomer might self-assemble into the spherical aggregates observed in the TEM micrographs (Figure 3c). To counterbalance the reduction of the *Z*-AZB population and the general depletion of BTA-AZB-GFLG monomer caused by the enzyme, the equilibrium is continuously shifted toward the nonassembled state until the degradation process is complete.

### Dual Stimuli Response: Cooperative Effect

To exploit this phenomenon, we evaluated the enzyme response of the fibers under continuous UV irradiation. The purpose of this assay was to test the cooperative application of the two stimuli (light and enzyme) led us to achieve a faster degradation time of BTA-AZB-GFLG fibers. The results clearly demonstrate the effectiveness of this approach, with nearly complete enzyme response after 1.5 h of continuous UV irradiation (Figure 4a,b). After 0.5 h, 55% of the enzyme response was achieved, which represents a 48-fold increase in reaction rate compared to the gate effect (UV irradiation for 10 min). TEM images were also acquired to further confirm the success of this cooperative approach (Figure 4c). In addition, a control experiment (under continuous UV light irradiation at 37 °C with no enzyme addition) was also studied by HPLC-MS (Figure S14). The results demonstrated that no degradation occurs in the absence of CTSB.

**Figure 4:**
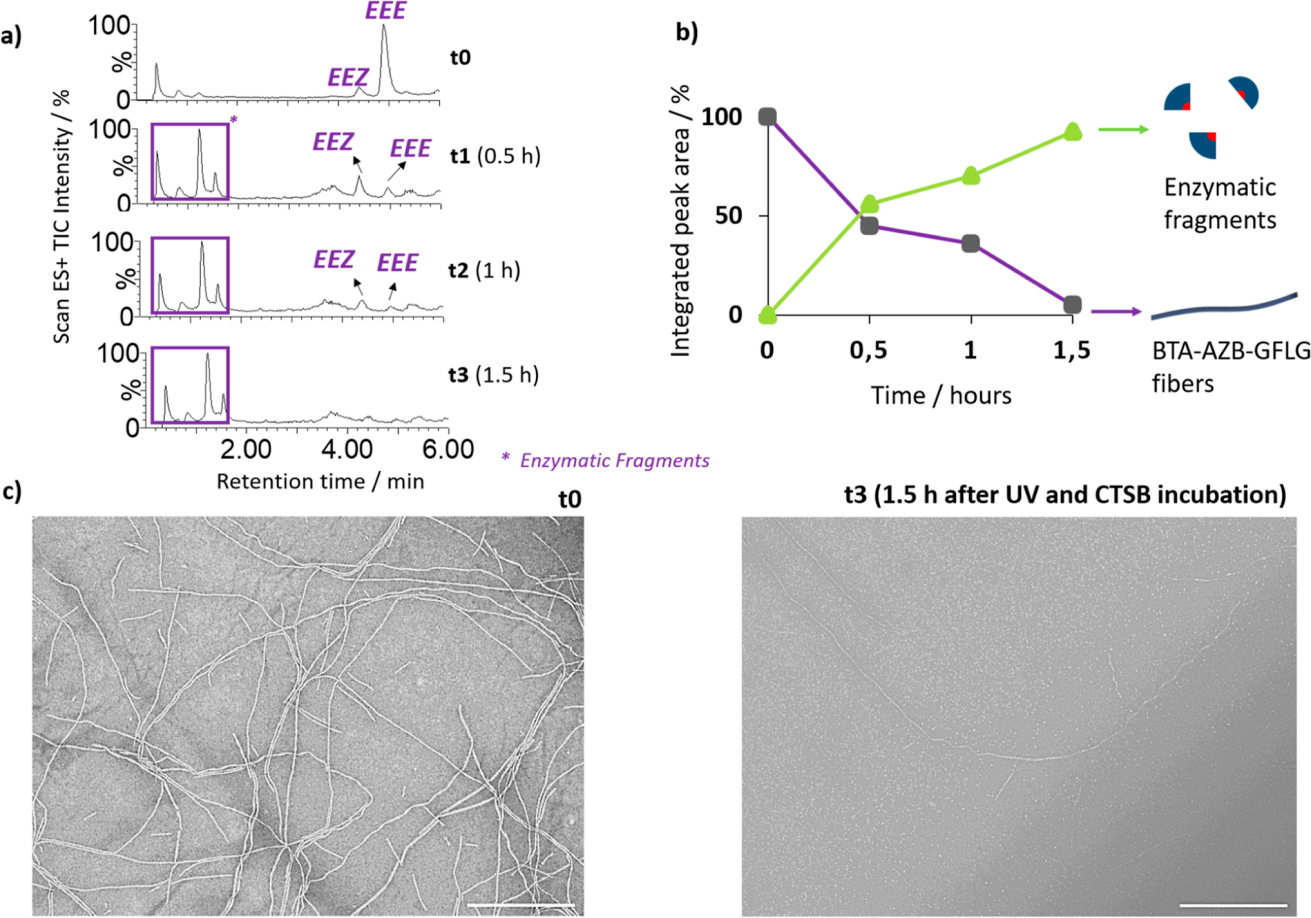
BTA-AZB-GFLG (25 μM) response to CTSB (3.8 U/mL) under continuous UV irradiation for 1.5 h (cooperative effect). HPLC-MS chromatograms (a) and integrated peak areas (b) following the enzymatic degradation. (c) Representative TEM micrographs (scale bar: 500 nm).

## Conclusion

One of the most interesting results of this research is the modularity of BTA-based monomers. In this work, we proved the possibility of building multifunctional BTAs by changing the amino acid sequence depending on the desired application. Our findings also demonstrate the possibility to catalyze the enzymatic response of BTA-AZB-GFLG fibers using UV light irradiation. That cooperative response can be attributed to the strategic location of GFLG in the monomer design. The sequence was located in the innermost region near the hydrophobic core, thereby limiting its accessibility to the CTSB and preventing undesired early hydrolysis. Moreover, a cooperative response was achieved by the synergistic use of light and enzyme stimuli. Notably, we developed a novel strategy to achieve selectivity over the enzyme responsiveness of the fibrillar state. By varying the light irradiation time, it would be possible to achieve either slow or fast release of entrapped molecules through gate or cooperative effects, respectively. Future prospects for this research include the integration of drugs in supramolecular fibers and the behavior of this novel system in cells. In this sense, our further research will focus on the use of alternative molecular photoswitches operating in the red or near-infrared region, which are more suitable for cellular applications.

## Supplementary Material



## Notes

Additional data and figures for the synthesis of BTA-AZB-GFLG, formulation and characterization of the supramolecular assemblies, and enzyme and light response assays (PDF)

## References

[1] Dong R., Zhou Y., Huang X., Zhu X., Lu Y., Shen J. (2015). Functional
Supramolecular Polymers for Biomedical Applications. Adv. Mater..

[2] Yan M., Wu S., Wang Y., Liang M., Wang M., Hu W., Yu G., Mao Z., Huang F., Zhou J. (2024). Recent Progress of
Supramolecular Chemotherapy Based on Host–Guest Interactions. Adv. Mater..

[3] Webber M. J., Langer R. (2017). Drug Delivery by Supramolecular
Design. Chem. Soc. Rev..

[4] Zhou J., Yu G., Huang F. (2017). Supramolecular Chemotherapy Based on Host–Guest
Molecular Recognition: A Novel Strategy in the Battle against Cancer
with a Bright Future. Chem. Soc. Rev..

[5] Bawa P., Pillay V., Choonara Y., du Toit L. (2009). Stimuli-Responsive
Polymers and Their Applications in Drug Delivery. Biomed. Mater..

[6] Cabane E., Zhang X., Langowska K., Palivan C. G., Meier W. (2012). Stimuli-Responsive
Polymers and Their Applications in Nanomedicine. Biointerphases.

[7] Mendes A. C., Baran E. T., Reis R. L., Azevedo H. S. (2013). Self-Assembly in
Nature: Using the Principles of Nature to Create Complex Nanobiomaterials. WIREs Nanomed. Nanobiotechnol..

[8] Krieg E., Bastings M. M. C., Besenius P., Rybtchinski B. (2016). Supramolecular
Polymers in Aqueous Media. Chem. Rev..

[9] Brunsveld L., Folmer B. J. B., Meijer E. W., Sijbesma R. P. (2001). Supramolecular Polymers. Chem.
Rev..

[10] Martínez-Orts M., Pujals S. (2024). Responsive Supramolecular Polymers for Diagnosis and
Treatment. Int. J. Mol. Sci..

[11] Dumitrache L. C., Shimada M., Downing S. M., Kwak Y. D., Li Y., Illuzzi J. L., Russell H. R., Wilson D. M., McKinnon P. J. (2018). Apurinic
Endonuclease-1 Preserves Neural Genome Integrity to Maintain Homeostasis
and Thermoregulation and Prevent Brain Tumors. Proc. Natl. Acad. Sci. U.S.A..

[12] Xiang J., Zhou L., Zhuang Y., Zhang J., Sun Y., Li S., Zhang Z., Zhang G., He Y. (2018). Lactate Dehydrogenase
Is Correlated with Clinical Stage and Grade and Is Downregulated by
si-SAΤB1 in Ovarian Cancer. Oncol. Rep..

[13] Sandoval J.
E., Huang Y.-H., Muise A., Goodell M. A., Reich N. O. (2019). Mutations
in the DNMT3A DNA Methyltransferase in Acute Myeloid Leukemia Patients
Cause Both Loss and Gain of Function and Differential Regulation by
Protein Partners. J. Biol. Chem..

[14] Xing X., Kane D. P., Bulock C. R., Moore E. A., Sharma S., Chabes A., Shcherbakova P. V. (2019). A Recurrent
Cancer-Associated Substitution
in DNA Polymerase ε Produces a Hyperactive Enzyme. Nat. Commun..

[15] Liu V. M., Howell A. J., Hosios A. M., Li Z., Israelsen W. J., Vander Heiden M. G. (2020). Cancer-Associated Mutations in Human
Pyruvate Kinase
M2 Impair Enzyme Activity. FEBS Lett..

[16] Xiao X., Huang J. (2024). Enzyme-Responsive Supramolecular
Self-Assembly in Small Amphiphiles. Langmuir.

[17] Li Q., Schenning A. P. H. J., Bunning T. J. (2019). Light-Responsive Smart Soft Matter
Technologies. Adv. Opt. Mater..

[18] Mura S., Nicolas J., Couvreur P. (2013). Stimuli-Responsive
Nanocarriers for
Drug Delivery. Nat. Mater..

[19] Roy N., Schädler V., Lehn J.-M. (2024). Supramolecular Polymers: Inherently
Dynamic Materials. Acc. Chem. Res..

[20] Lehn, J.-M. D.. From Supramolecular Polymers to Adaptive Dynamic Polymers. In Hierarchical Macromolecular Structures: 60 Years after the Staudinger Nobel Prize I; Percec, V. , Ed.; Springer International Publishing: Cham, 2013; pp 155–172.

[21] Qi G.-B., Gao Y.-J., Wang L., Wang H. (2018). Self-Assembled Peptide-Based
Nanomaterials for Biomedical Imaging and Therapy. Adv. Mater..

[22] Wang H., Mills J., Sun B., Cui H. (2024). Therapeutic Supramolecular
Polymers: Designs and Applications. Prog. Polym.
Sci..

[23] Zhang W., Wang F., Wang H., Xu T., Su H., Cui H. (2025). Balancing Chemical and Supramolecular
Stability in OEGylated Supramolecular
Polymers for Systemic Drug Delivery. J. Am.
Chem. Soc..

[24] Cheetham A. G., Zhang P., Lin Y., Lock L. L., Cui H. (2013). Supramolecular
Nanostructures Formed by Anticancer Drug Assembly. J. Am. Chem. Soc..

[25] Santini R., Fuentes E., Maleeva G., Matera C., Riefolo F., Berrocal J. A., Albertazzi L., Gorostiza P., Pujals S. (2025). Discotic Amphiphilic Supramolecular Polymers for Drug
Release and Cell Activation with Light. Nanoscale.

[26] Besenius P., Goedegebure Y., Driesse M., Koay M., Bomans P. H. H., Palmans A. R. A., Dankers P. Y. W., Meijer E. W. (2011). Peptide
Functionalised Discotic Amphiphiles and Their Self-Assembly into Supramolecular
Nanofibres. Soft Matter.

[27] BrunsveldLohmeijerVekemansMeijer L. B. G. G. J. A. J. M. E. W., Lohmeijer B. G., Vekemans M., Meijer W., Chirality E. (2000). Amplification in Dynamic Helical Columns in Water. Chem. Commun..

[28] Bochicchio D., Pavan G. (2018). Molecular Modelling of Supramolecular Polymers. Adv. Phys.:X.

[29] Wu B., Liu L., Zhou L., Magana J. R., Hendrix M. M. R. M., Wang J., Li C., Ding P., Wang Y., Guo X., Voets I. K., Cohen Stuart M. A., Wang J. (2022). Complex Supramolecular
Fiber Formed by Coordination-Induced Self-Assembly of Benzene-1,3,5-Tricarboxamide
(BTA). J. Colloid Interface Sci..

[30] Cantekin S., de Greef T. F. A., Palmans A. R. A. (2012). Benzene-1,3,5-Tricarboxamide:
A Versatile
Ordering Moiety for Supramolecular Chemistry. Chem. Soc. Rev..

[31] Leenders C. M. A., Albertazzi L., Mes T., Koenigs M. M. E., Palmans A. R. A., Meijer E. W. (2013). Supramolecular Polymerization in Water Harnessing Both
Hydrophobic Effects and Hydrogen Bond Formation. Chem. Commun..

[32] Leenders C. M. A., Mes T. B., Baker M., Koenigs M. M. E., Besenius P., Palmans A., Meijer E. W., Meijer W., From E. (2014). Supramolecular
Polymers to Hydrogel Materials. Mater. Horiz..

[33] Leenders C. M. A., Baker M. B., Pijpers I. A., Lafleur R. P. M., Albertazzi L., Palmans A., Meijer E. W., Meijer E. (2016). Supramolecular Polymerisation
in Water; Elucidating the Role of Hydrophobic and Hydrogen-Bond Interactions. Soft Matter.

[34] Bakker M. H., Lee C. C., Meijer E. W., Dankers P. Y. W., Albertazzi L. (2016). Multicomponent
Supramolecular Polymers as a Modular Platform for Intracellular Delivery. ACS Nano.

[35] Wijnands S. P. W., Engelen W., Lafleur R. P. M., Meijer E. W., Merkx M. (2018). Controlling
Protein Activity by Dynamic Recruitment on a Supramolecular Polymer
Platform. Nat. Commun..

[36] Hendrikse S. I. S., Su L., Hogervorst T. P., Lafleur R. P. M., Lou X., van der Marel G. A., Codee J. D. C., Meijer E. W. (2019). Elucidating the
Ordering in Self-Assembled Glycocalyx Mimicking Supramolecular Copolymers
in Water. J. Am. Chem. Soc..

[37] Morgese G., Waal B. F. M., Varela-Aramburu S., Palmans A. R. A., Albertazzi L., Meijer E. W. (2020). Anchoring Supramolecular
Polymers to Human Red Blood
Cells by Combining Dynamic Covalent and Non-Covalent Chemistries. Angew. Chem., Int. Ed..

[38] Varela-Aramburu S., Morgese G., Su L., Schoenmakers S. M. C., Perrone M., Leanza L., Perego C., Pavan G. M., Palmans A. R. A., Meijer E. W. (2020). Exploring the Potential of Benzene-1,3,5-Tricarboxamide
Supramolecular Polymers as Biomaterials. Biomacromolecules.

[39] Besenius P., Portale G., Bomans P. H. H., Janssen H. M., Palmans A. R. A., Meijer E. W. (2010). Controlling the
Growth and Shape of Chiral Supramolecular
Polymers in Water. Proc. Natl. Acad. Sci. U.S.A..

[40] Zuo C., Dai X., Zhao S., Liu X., Ding S., Ma L., Liu M., Wei H. (2016). Fabrication
of Dual-Redox Responsive Supramolecular
Copolymers Using a Reducible β-Cyclodextran-Ferrocene Double-Head
Unit. ACS Macro Lett..

[41] Lu Y., Zou H., Yuan H., Gu S., Yuan W., Li M. (2017). Triple Stimuli-Responsive
Supramolecular Assemblies Based on Host-Guest Inclusion Complexation
between β-Cyclodextrin and Azobenzene. Eur. Polym. J..

[42] Zhou Z., Li G., Wang N., Guo F., Guo L., Liu X. (2018). Synthesis
of Temperature/pH Dual-Sensitive Supramolecular Micelles from β-Cyclodextrin-Poly­(N-Isopropylacrylamide)
Star Polymer for Drug Delivery. Colloids Surf.
B Biointerfaces..

[43] Wei P., Sun M., Yang B., Xiao J., Du J. (2020). Ultrasound-Responsive
Polymersomes Capable of Endosomal Escape for Efficient Cancer Therapy. J. Controlled Release.

[44] Kawano S., Lie J., Ohgi R., Shizuma M., Muraoka M. (2021). Modulating Polymeric
Amphiphiles Using Thermo- and pH-Responsive Copolymers with Cyclodextrin
Pendant Groups through Molecular Recognition of the Lipophilic Dye. Macromolecules.

[45] Fuentes E., Gerth M., Berrocal J. A., Matera C., Gorostiza P., Voets I. K., Pujals S., Albertazzi L. (2020). An Azobenzene-Based
Single-Component Supramolecular Polymer Responsive to Multiple Stimuli
in Water. J. Am. Chem. Soc..

[46] Seymour L. W., Ferry D. R., Anderson D., Hesslewood S., Julyan P. J., Poyner R., Doran J., Young A. M., Burtles S., Kerr D. J. (2002). Cancer Research Campaign Phase I/II
Clinical Trials committee. Hepatic Drug Targeting: Phase I Evaluation
of Polymer-Bound Doxorubicin. J. Clin. Oncol..

[47] Rademaker-Lakhai J. M., Terret C., Howell S. B., Baud C. M., De Boer R. F., Pluim D., Beijnen J. H., Schellens J. H. M., Droz J.-P. (2004). A Phase I and Pharmacological Study
of the Platinum
Polymer AP5280 given as an Intravenous Infusion Once Every 3 Weeks
in Patients with Solid Tumors. Clin. Cancer
Res..

[48] Meerum
Terwogt J. M., ten Bokkel Huinink W.
W., Schellens J. H., Schot M., Mandjes I. A., Zurlo M. G., Rocchetti M., Rosing H., Koopman F. J., Beijnen J. H. (2001). Phase I Clinical
and Pharmacokinetic Study of PNU166945, a Novel Water-Soluble Polymer-Conjugated
Prodrug of Paclitaxel. Anticancer Drugs.

[49] Zhang C., Pan D., Li J., Hu J., Bains A., Guys N., Zhu H., Li X., Luo K., Gong Q., Gu Z. (2017). Enzyme-Responsive
Peptide Dendrimer-Gemcitabine Conjugate as a Controlled-Release Drug
Delivery Vehicle with Enhanced Antitumor Efficacy. Acta Biomater..

[50] Gondi C. S., Rao J. S. (2013). Cathepsin B as a
Cancer Target. Expert Opin. Ther. Targets.

[51] Wu Z., Ni J., Liu Y., Teeling J. L., Takayama F., Collcutt A., Ibbett P., Nakanishi H. (2017). Cathepsin B Plays a Critical Role
in Inducing Alzheimer’s Disease-like Phenotypes Following Chronic
Systemic Exposure to Lipopolysaccharide from *Porphyromonas
Gingivalis* in Mice. Brain, Behav.,
Immun..

[52] Hook V., Yoon M., Mosier C., Ito G., Podvin S., Head B. P., Rissman R., O’Donoghue A. J., Hook G. (2020). Cathepsin B in Neurodegeneration of Alzheimer’s Disease, Traumatic
Brain Injury, and Related Brain Disorders. Biochim.
Biophys Acta Proteins Proteom..

[53] Siddiqui A. A., Merquiol E., Bruck-Haimson R., Hirbawi J., Boocholez H., Cohen I., Yan Y., Dong M. Q., Blum G., Cohen E. (2024). Cathepsin B Promotes Aβ Proteotoxicity by Modulating Aging
Regulating Mechanisms. Nat. Commun..

[54] Howie A. J., Burnett D., Crocker J. (1985). The Distribution of
Cathepsin B in
Human Tissues. J. Pathol..

[55] Vidak E., Javoršek U., Vizovišek M., Turk B. (2019). Cysteine Cathepsins
and Their Extracellular Roles: Shaping the Microenvironment. Cells.

[56] Ni J., Lan F., Xu Y., Nakanishi H., Li X. (2022). Extralysosomal Cathepsin
B in Central Nervous System: Mechanisms and Therapeutic Implications. Brain Pathol..

[57] Li W., Park I., Kang S.-K., Lee M. (2012). Smart Hydrogels from
Laterally-Grafted Peptide Assembly. Chem. Commun..

[58] Bose M., Groff D., Xie J., Brustad E., Schultz P. G. (2006). The Incorporation
of a Photoisomerizable Amino Acid into Proteins in E. Coli. J. Am. Chem. Soc..

[59] Fmoc Solid Phase Peptide Synthesis: A Practical Approach; Chan, W. C. , White, P. D. , Eds.; Oxford University Press: New York, 2020.

[60] Schneider C. A., Rasband W. S., Eliceiri K. W. (2012). NIH Image to ImageJ: 25 Years of
Image Analysis. Nat. Methods.

[61] Xie Z., Zhao M., Yan C., Kong W., Lan F., Zhao S., Yang Q., Bai Z., Qing H., Ni J. (2023). Cathepsin B in Programmed Cell Death Machinery: Mechanisms of Execution
and Regulatory Pathways. Cell Death Dis..

[62] Jin X., Zhang J., Jin X., Liu L., Tian X. (2020). Folate Receptor
Targeting and Cathepsin B-Sensitive Drug Delivery System for Selective
Cancer Cell Death and Imaging. ACS Med. Chem.
Lett..

[63] Liu M., Zhang L., Wang T. (2015). Supramolecular Chirality in Self-Assembled
Systems. Chem. Rev..

[64] Núñez I., Merino E., Lecea M., Pieraccini S., Spada G. P., Rosini C., Mazzeo G., Ribagorda M., Carreño M. C. (2013). Control of the Helical Chirality of Enantiopure Sulfinyl
(Z)-Azobenzene-Based Photoswitches. Chem.—Eur.
J..

[65] Schoenmakers S. M. C., Spiering A. J. H., Herziger S., Böttcher C., Haag R., Palmans A. R. A., Meijer E. W. (2022). Structure
and Dynamics
of Supramolecular Polymers: Wait and See. ACS
Macro Lett..

[66] Zhang W., Xie J., Yang Z., Shi W. (2007). Aggregation Behaviors and Photoresponsive
Properties of Azobenzene Constructed Phosphate Dendrimers. Polymer.

[67] Leung, F. K.-C. Aqueous Supramolecular Assemblies of Photocontrolled Molecular Amphiphiles. In Supramolecular Assemblies Based on Electrostatic Interactions; Aboudzadeh, M. A. , Frontera, A. , Eds.; Springer International Publishing: Cham, 2022; pp 267–308.

[68] Pianowski Z. L. (2019). Recent
Implementations of Molecular Photoswitches into Smart Materials and
Biological Systems. Chem.—Eur. J..

[69] Adhikari B., Yamada Y., Yamauchi M., Wakita K., Lin X., Aratsu K., Ohba T., Karatsu T., Hollamby M. J., Shimizu N., Takagi H., Haruki R., Adachi S., Yagai S. (2017). Light-Induced Unfolding
and Refolding of Supramolecular Polymer Nanofibres. Nat. Commun..

[70] Szymański W., Beierle J. M., Kistemaker H. A. V., Velema W. A., Feringa B. L. (2013). Reversible
Photocontrol of Biological Systems by the Incorporation of Molecular
Photoswitches. Chem. Rev..

[71] Xu F., Feringa B. L. (2023). Photoresponsive Supramolecular Polymers: From Light-Controlled
Small Molecules to Smart Materials. Adv. Mater..

[72] Cheung L.-H., Kajitani T., Leung F. K.-C. (2022). Visible-Light
Controlled Supramolecular
Transformations of Donor-Acceptor Stenhouse Adducts Amphiphiles at
Multiple Length-Scale. J. Colloid Interface
Sci..

[73] Endo M., Fukui T., Jung S. H., Yagai S., Takeuchi M., Sugiyasu K. (2016). Photoregulated Living
Supramolecular Polymerization
Established by Combining Energy Landscapes of Photoisomerization and
Nucleation-Elongation Processes. J. Am. Chem.
Soc..

[74] Pal D. S., Kar H., Ghosh S. (2016). Phototriggered Supramolecular Polymerization. Chem.—Eur. J..

[75] Ren Y., Zhou Z., Harley I., Aydin O. ¨., Driehaus-Ortiz L., Kaltbeitzel A., Xing J., Maxeiner K., Lieberwirth I., Landfester K., Ng D. Y. W., Weil T. (2025). Intracellular
Assembly of Supramolecular Peptide Nanostructures Controlled by Visible
Light. Nat. Synth..

[76] Tao Y., Chan H. F., Shi B., Li M., Leong K. W. (2020). Light:
A Magical Tool for Controlled Drug Delivery. Adv. Funct. Mater..

[77] Bandara H. M. D., Burdette S. C. (2012). Photoisomerization
in Different Classes of Azobenzene. Chem. Soc.
Rev..

[78] Beharry A. A., Woolley G. A. (2011). Azobenzene Photoswitches
for Biomolecules. Chem. Soc. Rev..

[79] Titov E., Granucci G., Götze J. P., Persico M., Saalfrank P. (2016). Dynamics of
Azobenzene Dimer Photoisomerization: Electronic and Steric Effects. J. Phys. Chem. Lett..

[80] Cocchi C., Draxl C. (2017). Understanding the Effects of Packing
and Chemical Terminations on
the Optical Excitations of Azobenzene-Functionalized Self-Assembled
Monolayers. J. Phys.: Condens. Matter..

[81] Cheng H.-B., Zhang S., Qi J., Liang X.-J., Yoon J. (2021). Advances in
Application of Azobenzene as a Trigger in Biomedicine: Molecular Design
and Spontaneous Assembly. Adv. Mater..

