# Supplementary material for: Light and Enzymatic Cooperative Response in Supramolecular Fibers: A Synergistic Strategy for Potential Drug Delivery Applications
Source: Biomacromolecules. 2026 Apr 17;27(5):3376–84. doi: 10.1021/acs.biomac.6c00354 (PMC13169339; doi:10.1021/acs.biomac.6c00354)
Supplement: Supplementary file 1 [file bm6c00354_si_001.pdf]

Supporting Information (SI)

Light- and enzymatic cooperative response in supramolecular fibers: a novel strategy for potential drug delivery applications

Mónica Martínez-Orts, Edgar Fuentes, Yeray Gabaldón, José Augusto Berrocal, Lorenzo Albertazzi and Silvia Pujals*

Contents

1 Chemical Synthesis 2

2 Supramolecular assembly 3

2.1 Self-assembly characterization 3

3 Enzyme and light response 5

3.1 Enzymatic Response 5

3.2 Fibrillar stacking 8

3.3 Light response 9

3.4 Dual enzyme and light response 11

1 Chemical Synthesis

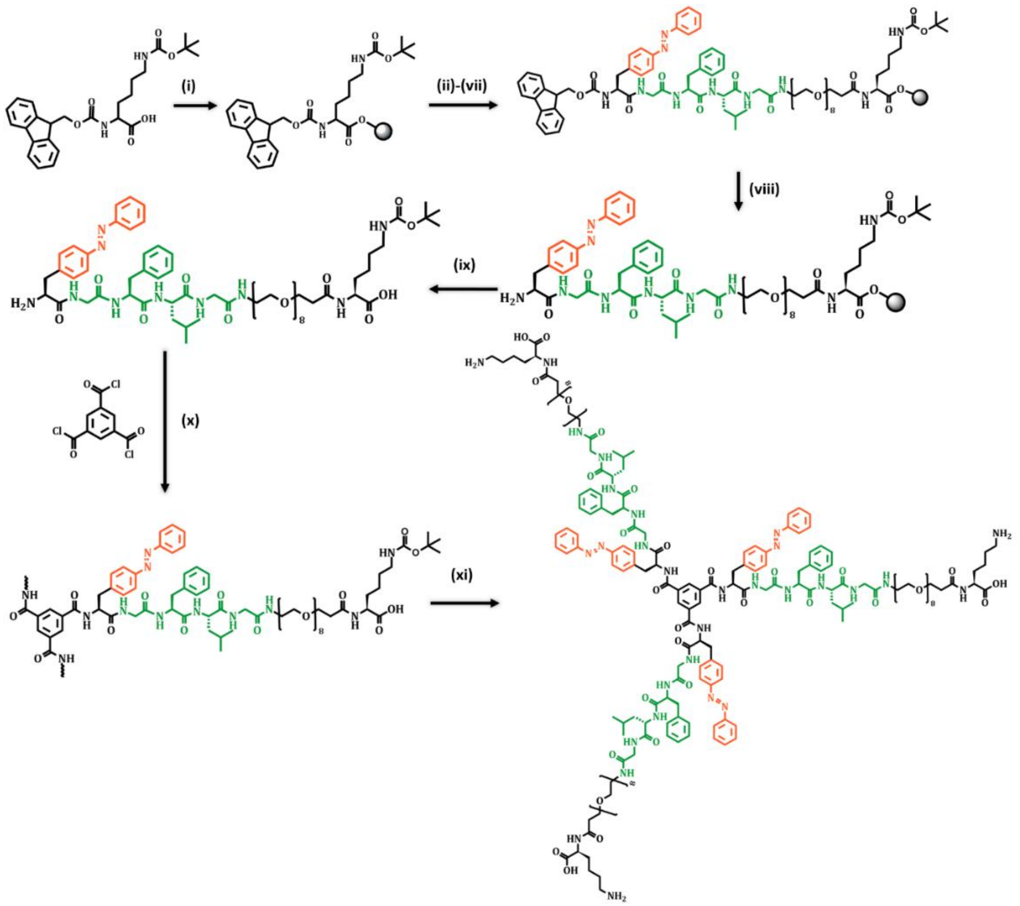

Scheme S1. Synthesis of BTA-AZB-GFLG monomer.

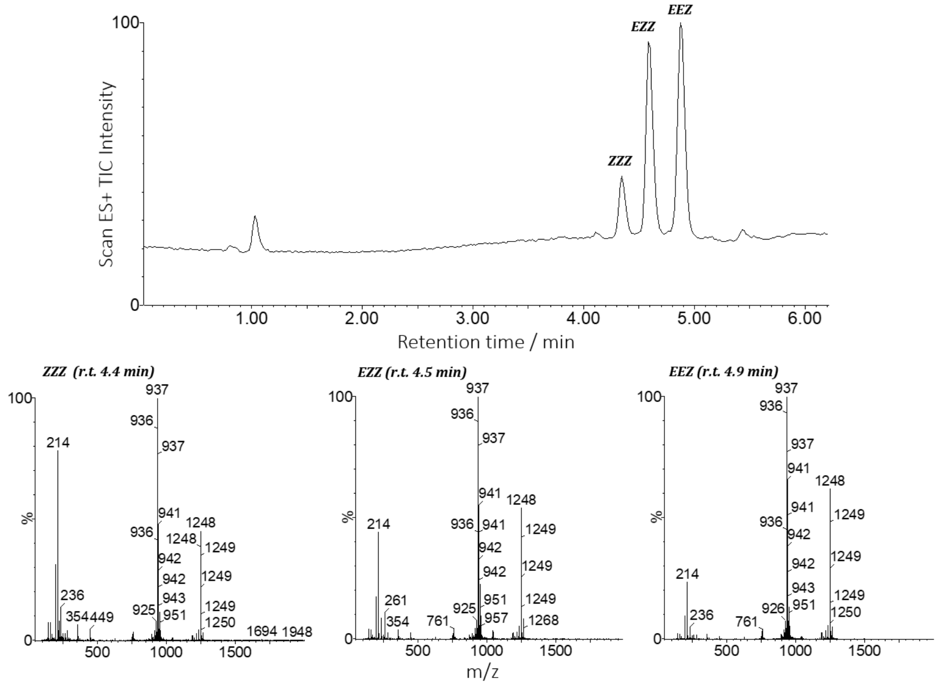

Figure S1. MALDI-TOF-MS of BTA-AZB-GFLG monomer (M.W. 3742.3 g/mol). Calculated m/z: $[M+3H]^{3+}$= 1248.4, $[M+4H]^{4+}$= 936.6. Experimental m/z: $[M+3H]^{3+}$= 1248, $[M+4H]^{4+}$= 937.

2 Supramolecular assembly

2.1 Self-assembly characterization

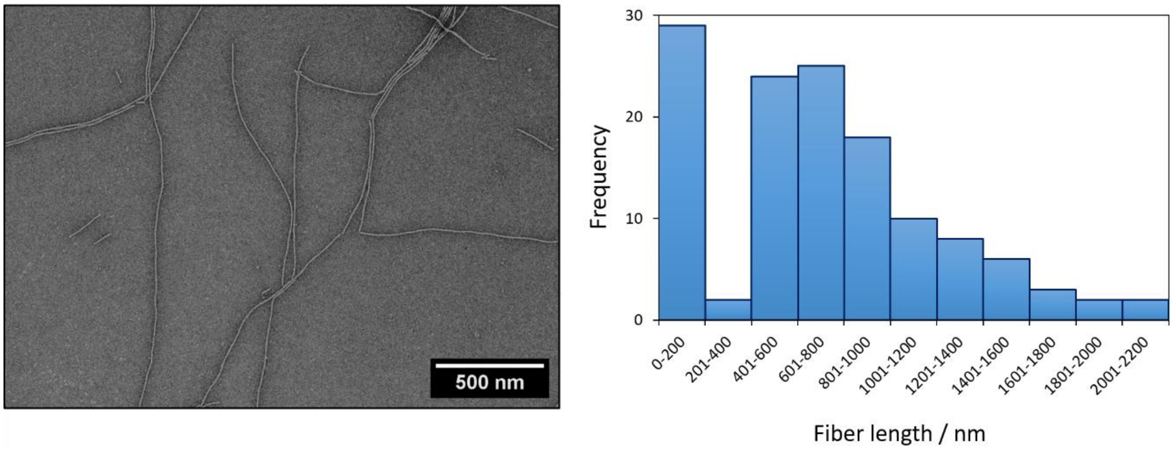

Figure S2. (Left) Representative TEM image of BTA-AZB-GFLG supramolecular fibers (25 μM) in water. Scale bar: 500 nm. (Right) Data histogram gathering the length (n = 126). Mean: 538.6; Median= 713.4; St. Dev. =478.9.

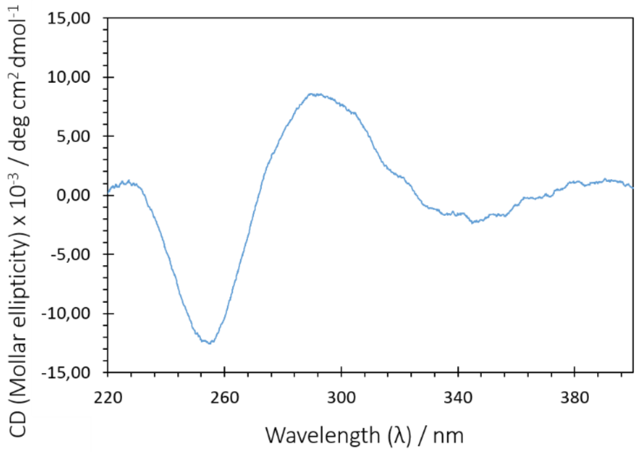

Figure S3. CD spectra of BTA-AZB-GFLG self-assembly in water (25 μM).

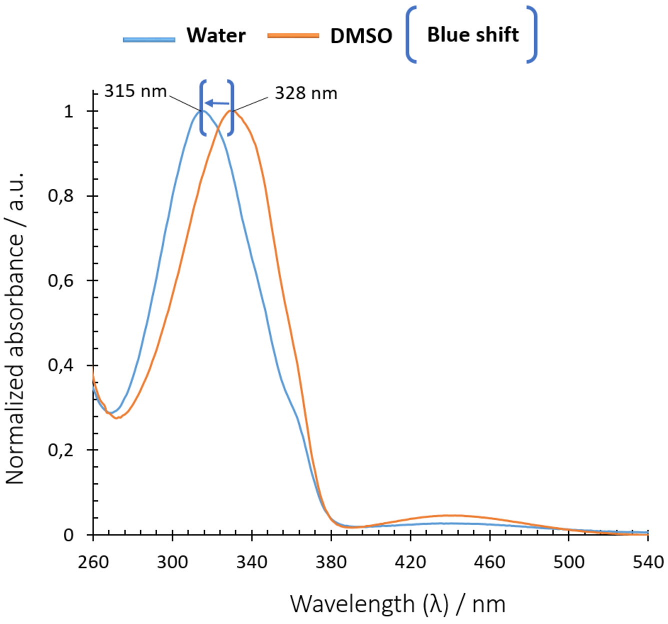

Figure S4. UV-Vis spectra of BTA-AZB-GFLG monomer in the non-assembled state (orange) compared to the assembled fibers (blue). The blue shift detected in the maximum absorbance peak is highlighted.

3 Enzyme and light response

3.1 Enzymatic Response

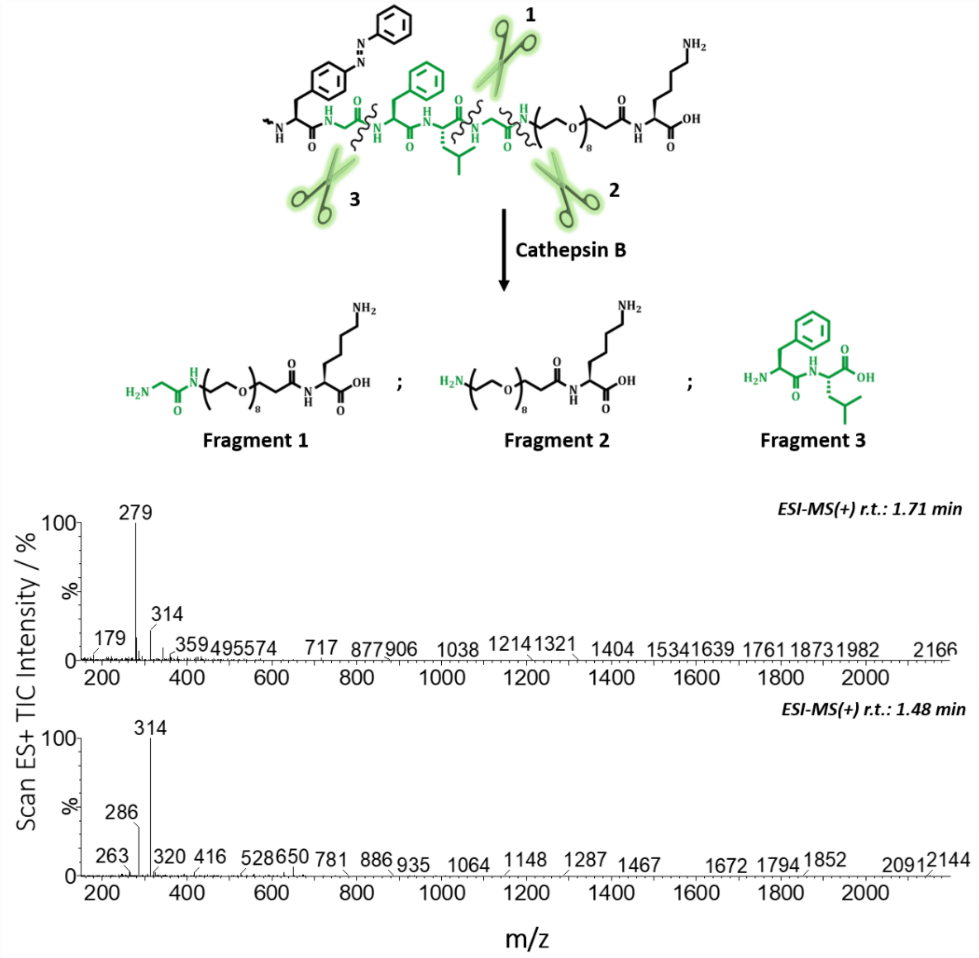

Scheme S2. Cleavage of GFLG by CTSB. Fragment 1 (retention time: 1.48 min; M.W. 626.74 g/mol; $[M+2H]^{2+}$ *m/z:* 314.37, experimental: 314); fragment 2 (retention time: 1.48 min; M.W. 569.69 g/mol; $[M+2H]^{2+}$ *m/z:* 285.85, experimental: 286); fragment 3 (retention time: 1.71 min; M.W. 278.35 g/mol; $[M+2H]^{2+}$ *m/z:* 279.35, experimental: 279). Representative ES-MS(+) of the enzymatic degradability of BTA-AZB-GFLG fibers 96 hours after the addition of CTSB, following prior UV irradiation.

a)

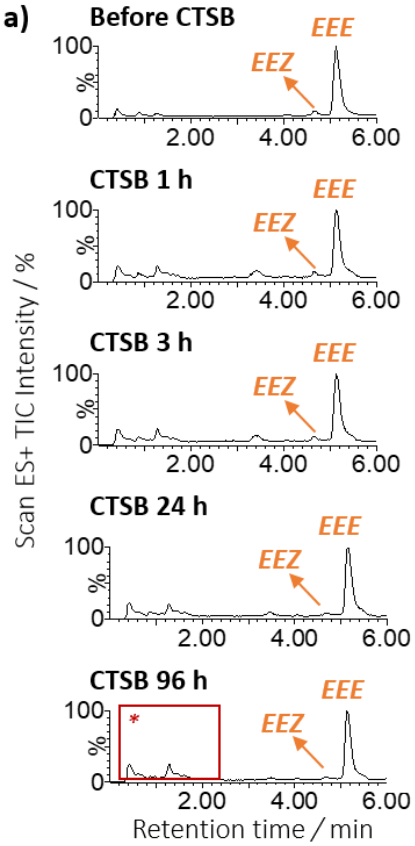

b)

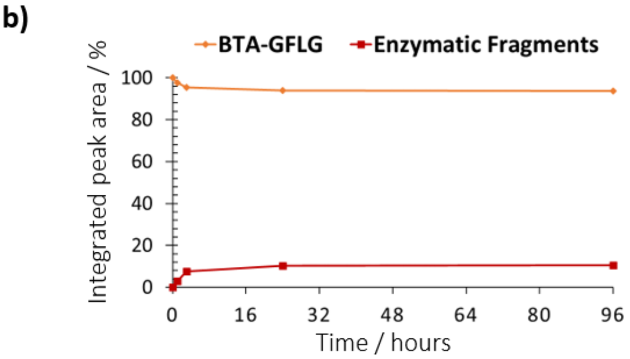

Incubation time with CTSB (hours)	BTA-AZB-GFLG % (Control, no UV)	Enzymatic Fragments % (Control, no UV)
0	100,0	0,0
1	92,9	2,7
3	90,6	7,1
24	89,2	9,8
96	89,1	9,9

** Enzymatic Fragments*

c)

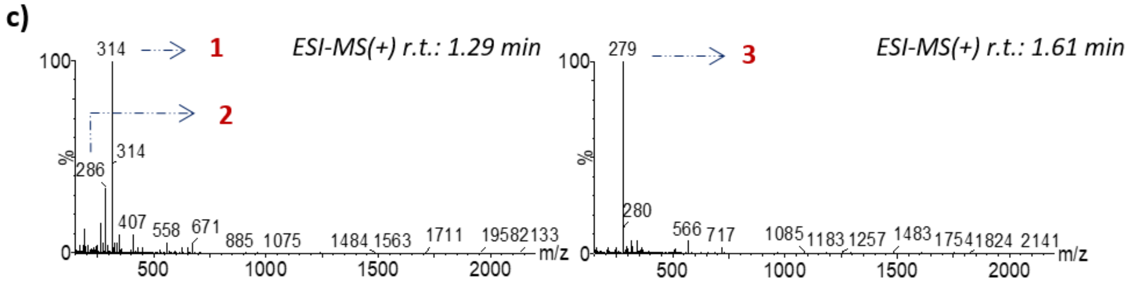

1 2 3

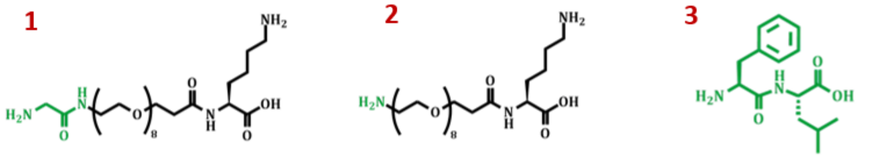

Figure S5. (a) HPLC-MS EF10 25 μM with CTSB 3.8 U/mL. **(b)** Kinetic study, normalized to EEE isomer (100 %), of the enzymatic degradation of BTA-AZB-GFLG (orange) and the presence of hydrolysis fragments (dark red) **(c)** ES (+)-MS of hydrolysis fragment 1 (M.W.: 626.74 g/mol; calculated m/z $[M+2H]^{2+}$= 314.4; experimental m/z= 314), hydrolysis fragment 2 (M.W.: 569.69 g/mol; calculated m/z $[M+2H]^{2+}$= 285.8, experimental m/z= 286), hydrolysis fragment 3 (M.W. 278.35 g/mol; $[M+2H]^{2+}$ *m/z:* 279.35, experimental: 279).

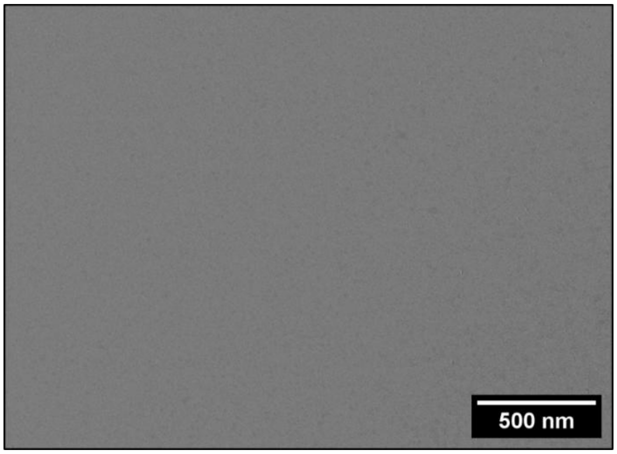

Figure S6. Transmission Electron Microscopy (TEM) image of Cathepsin B in absence of the BTA-AZB-GFLG substrate. Scale bar: 500 nm.

	**Frequency**		
**Fibers length (nm)**	*Before CTSB*	*24 h incubation*	*96 h incubation*
< 200	49	16	9
> 200	91	89	87

Table S1. Quantification of TEM images regarding the enzyme response of BTA-AZB-GFLG fibers with no UV (see Figure S2 in the main document).

3.2 Fibrillar stacking

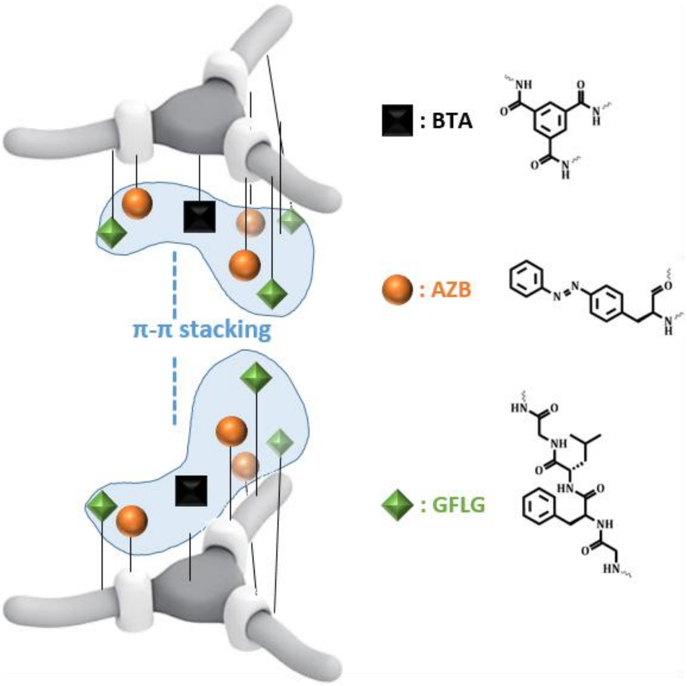

Figure S7. Hypothetical proposal regarding the strong fibrillar packaging of BTA-AZB-GFLG supramolecular fibers.

3.3 Light response

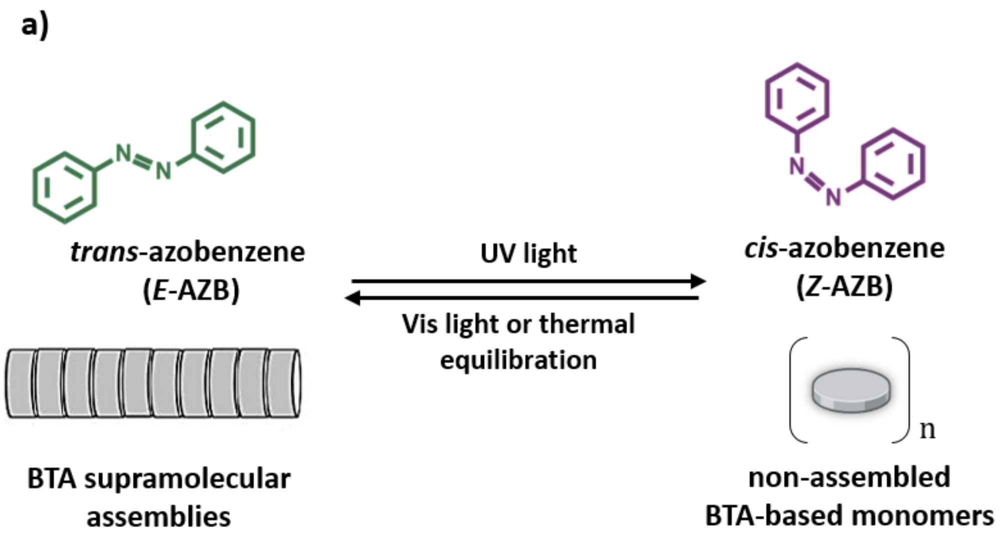

Figure S8. Cis-to-trans photoisomerization equilibrium of azobenzene switch is related to the assembly/disassembly of supramolecular fibers.

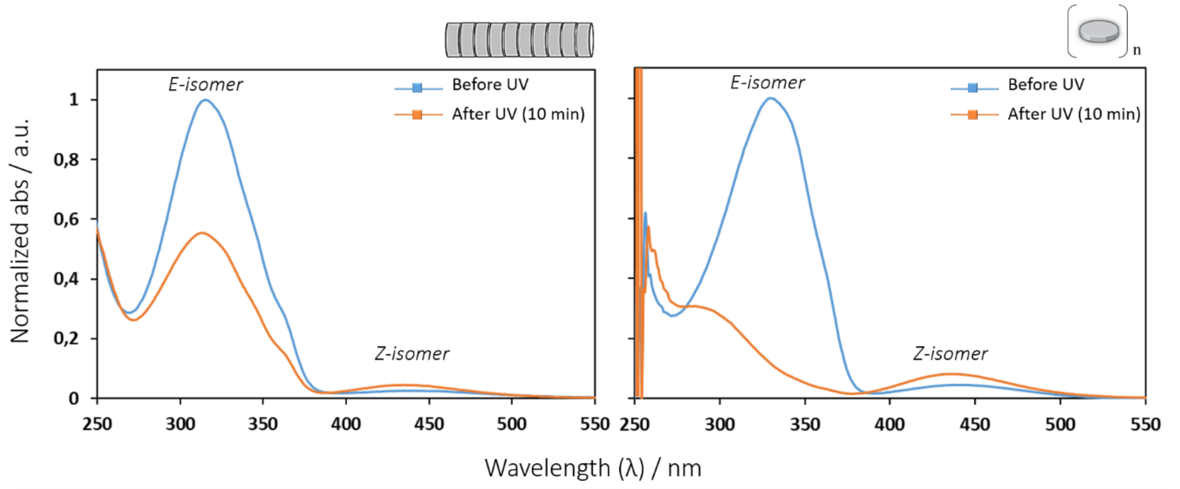

Figure S9. Light response in the self-assembled state evaluated in water (left) compared to the non-assembled monomer, in DMSO, (right). This experiment was performed by irradiating 25 μM solutions.

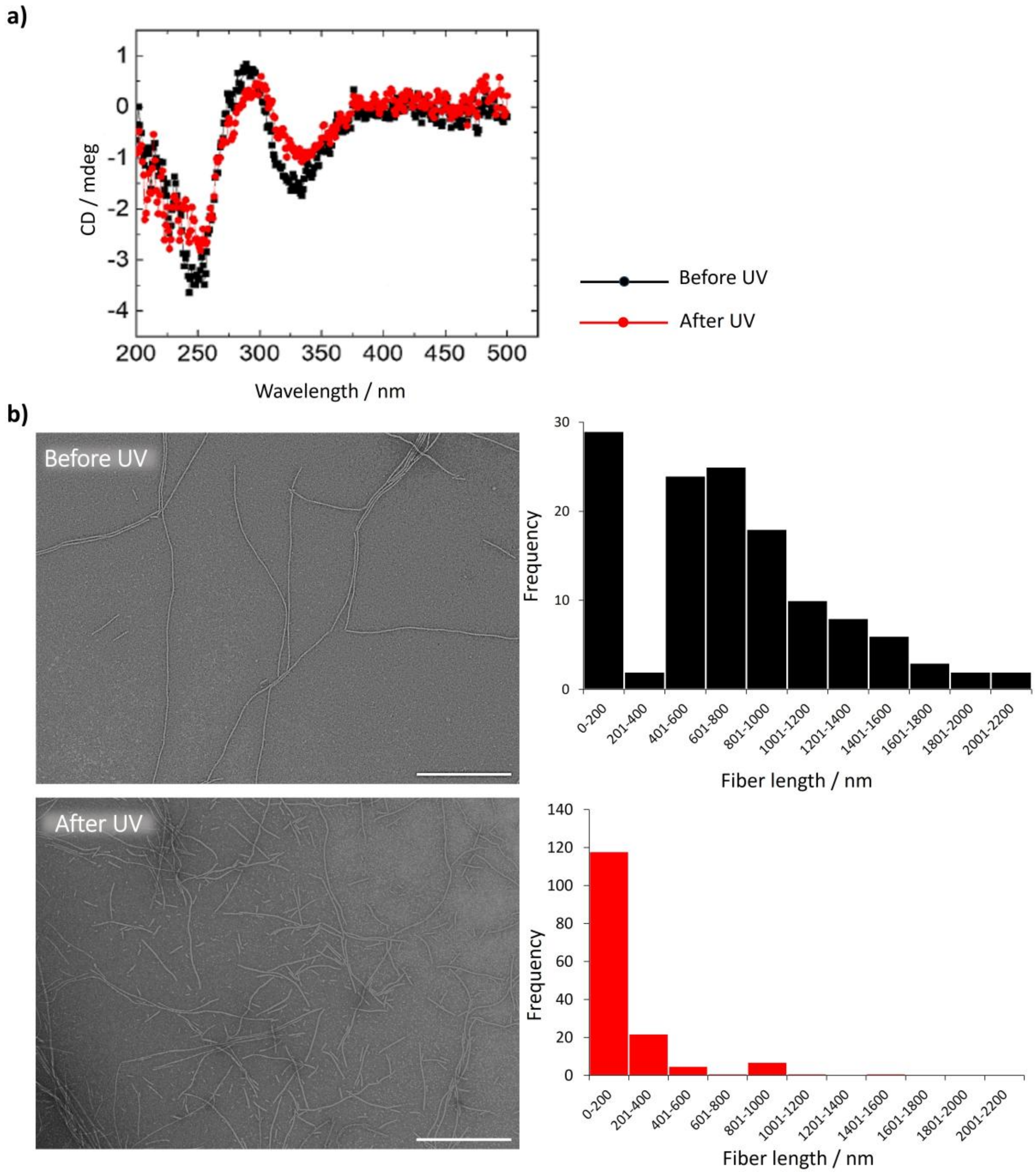

Figure S10. Photoresponse of BTA-AZB-GFLG supramolecular fibers at 25 μM in water upon UV irradiation (365 nm, 10 min, 100 % at 1000 mA of the LED intensity). **(a)** (Left) TEM images of the sample before and after UV irradiation (scale bar: 500 nm). (Right) histogram showing the length distribution of the fibers measured from TEM images before (black, n = 129) and after (red, n = 155) irradiation with UV light (n = total number of measured fibers). **(b)** CD spectra before (black) and after (red) irradiation with UV.

3.4 Dual enzyme and light response

Incubation time with CTSB (hours)	BTA-AZB-GFLG % (Control, no UV)	Enzymatic Fragments % (Control, no UV)	BTA-AZB-GFLG % (UV)		Enzymatic Fragments % (UV)	
0	100,0	0,0	Before UV	100,0	Before UV	0,0
0			After UV	100,0	After UV	0,0
1	92,9	2,7	58,1		39,8	
3	90,6	7,1	55,8		44,8	
24	89,2	9,8	52,0		47,7	
96	89,1	9,9	0,1		93,8	

Figure S11. Integrated peak area (as a percentage, with the total BTA-AZB-GFLG monomer set to 100 %) calculated from the HPLC-MS chromatograms shown in Figure 3a. In the case of the control experiment, without pre-UV, (BTA-AZB-GFLG degradation is colored inorange, while the enzymatic fragments are highlighted inblack), compared to the pre-irradiation with UV light (blue for the case of the BTA-AZB-GFLG degradation and dark red representing the enzymatic fragments). The calculated percentages are collected in the attached table.

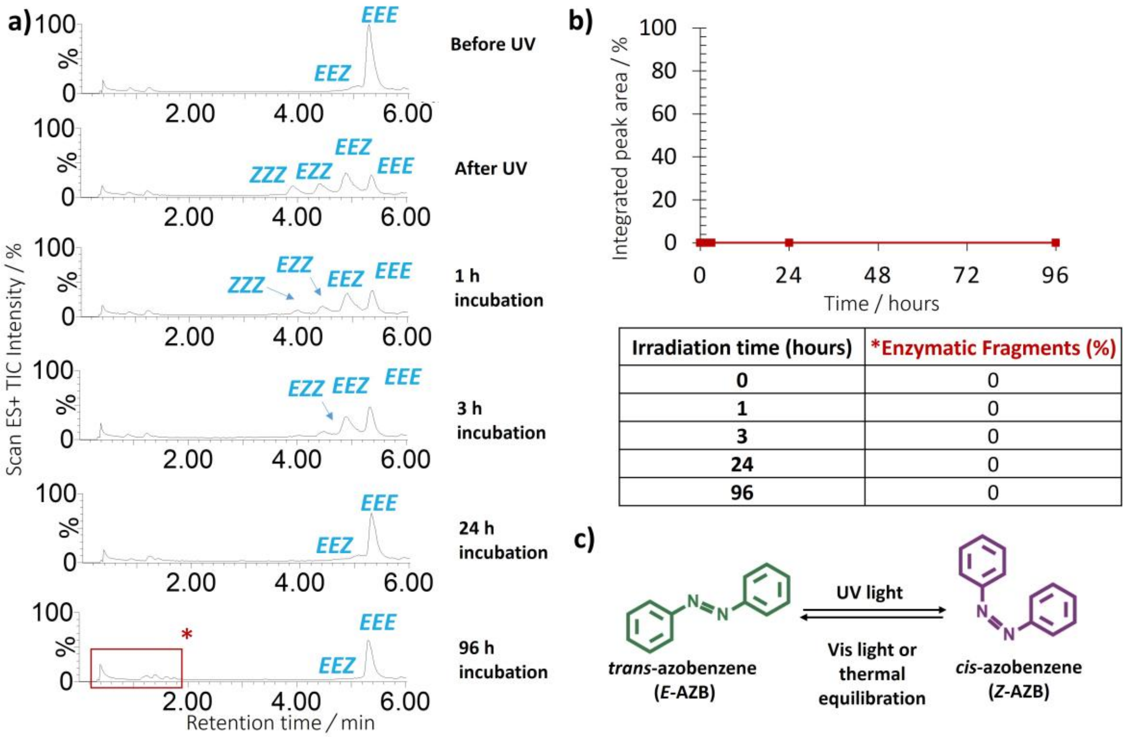

Figure S12. Thermal relaxation of *Z*-AZB (obtained after pre-UV irradiation for 10 minutes) in BTA-AZB-GFLG (25 µM) fibers during 96 hours at 37 °C. **(a)** HPLC-MS chromatograms at different incubation times in the absence of CTSB. **(b)** Graphical representation of the integrated HPLC-MS peak areas of the enzymatic fragments region. **(c)** Schematic explanation of *Z*-AZB thermal relaxation back to the *E*-AZB configuration.

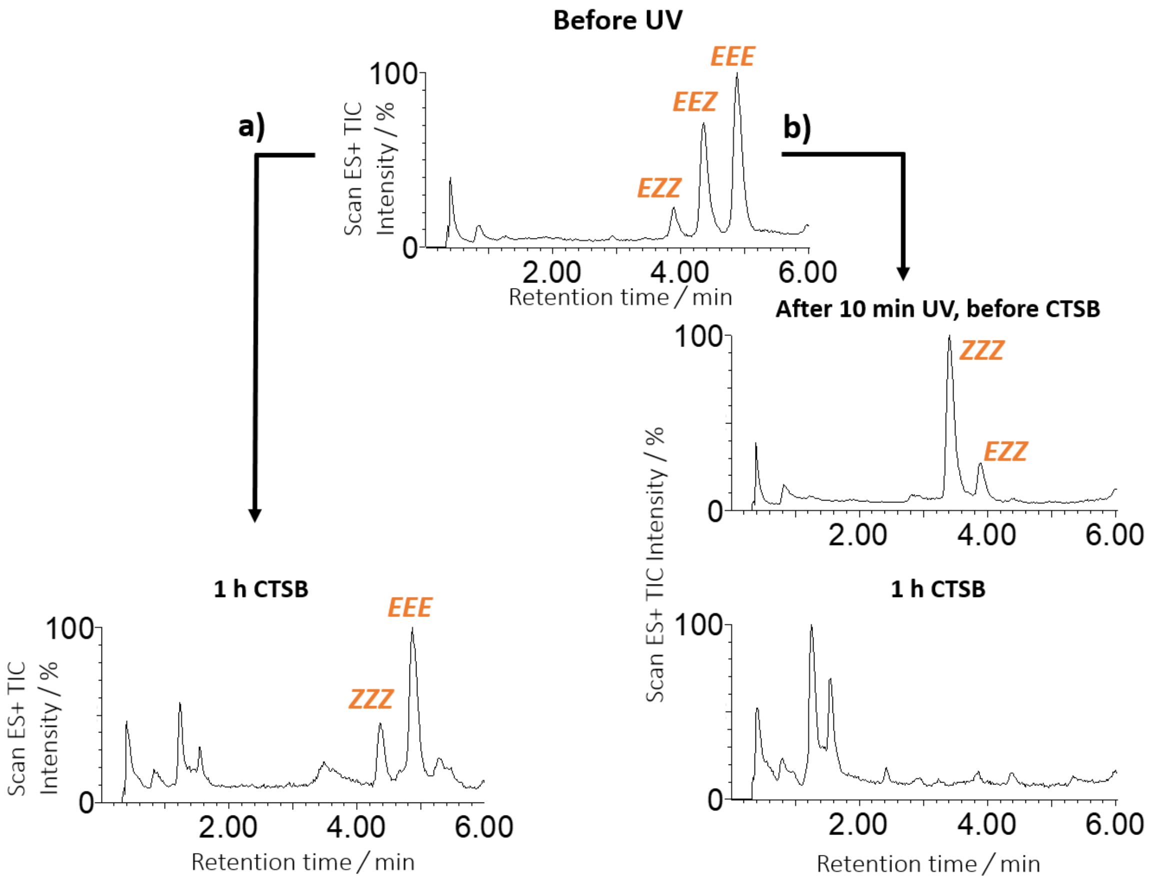

Figure S13. Enzymatic degradability assay in the initial assembly state without light stimulation (non-irradiated sample, **a**) and with prior irradiation with UV light (**b**).

The enzymatic assay of the initial assembly state slightly differs from that regarding the supramolecular fibers. In this case, the BTA-AZB-GFLG monomer (in DMSO) was directly injected into a buffer mixture that already contained Cathepsin B. The final concentration of BTA-AZB-GFLG was 25 μM and 3.8 U/mL in the case of the enzyme. The solvent used for this experiment was DTT buffer (pH = 5.0 ± 0.1). In the case of the assay with pre-UV light irradiation, this was directly applied to the DMSO stock.

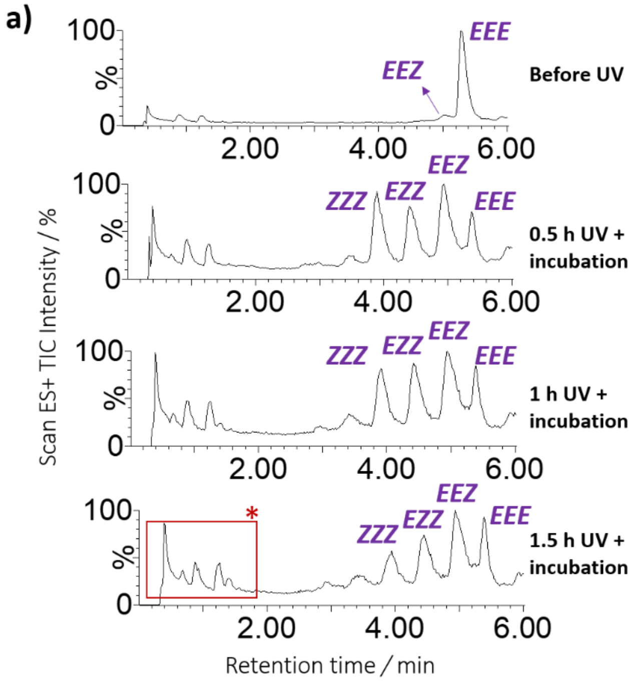

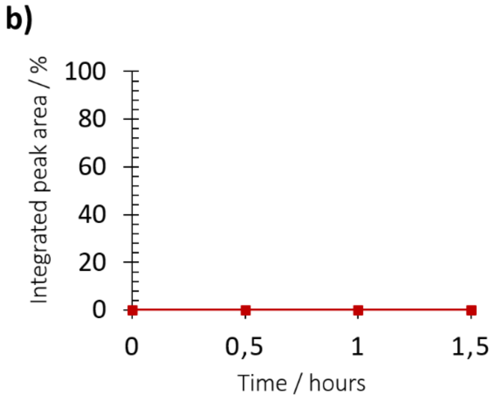

Irradiation time (hours)	*Enzymatic Fragments (%)
**0**	0
**0,5**	0
**1**	0
**1,5**	0

Figure S14. UV light-response evaluation of AZB motif in BTA-AZB-GFLG (25 µM) fibers during 1.5 hours at 37 °C. **(a)** HPLC-MS chromatograms at different incubation times in the absence of CTSB. **(b)** Graphical representation of the integrated HPLC-MS peak areas of the enzymatic fragments region.
